# Seasonal phytoplankton and geochemical shifts in the subsurface chlorophyll maximum layer of a dimictic ferruginous lake

**DOI:** 10.1002/mbo3.1287

**Published:** 2022-05-15

**Authors:** Elizabeth D. Swanner, Marina Wüstner, Tania Leung, Jürgen Pust, Micah Fatka, Nick Lambrecht, Hannah E. Chmiel, Harald Strauss

**Affiliations:** ^1^ Department of Geological & Atmospheric Sciences Iowa State University Ames Iowa USA; ^2^ Center for Applied Geoscience University of Tübingen Tübingen Germany; ^3^ Naturschutzgebietes Heiliges Meer Landschaftsverband Westfalen‐Lippe (LWL) Museum für Naturkunde Recke Germany; ^4^ Environmental Engineering Institute École Polytechnique Fédérale de Lausanne Lausanne Switzerland; ^5^ Institute for Geology and Paleontology University of Münster Münster Germany

**Keywords:** 16S and 18S rRNA gene sequencing, chemocline, iron, nutrient, photosynthesis, primary productivity, stratification

## Abstract

Subsurface chlorophyll maxima layers (SCML) are ubiquitous features of stratified aquatic systems. Availability of the micronutrient iron is known to influence marine SCML, but iron has not been explored in detail as a factor in the development of freshwater SCML. This study investigates the relationship between dissolved iron and the SCML within the dimictic, ferruginous lake Grosses Heiliges Meer in northern Germany. The occurrence of the SCML under nonferruginous conditions in the spring and ferruginous conditions in the fall are context to explore temporal changes in the phytoplankton community and indicators of primary productivity. Results indicate that despite more abundant chlorophyll in the spring, the SCML sits below a likely primary productivity maximum within the epilimnion, inferred based on colocated dissolved oxygen, δ^13^C_DIC_, and pH maxima. The peak amount of chlorophyll in the SCML is lower in the fall than in the spring, but in the fall the SCML is colocated with elevated dissolved iron concentrations and a local δ^13^C_DIC_ maximum. Cyanobacteria and Chlorophyta have elevated abundances within the SCML in the fall. Further investigation of the relationship of iron to primary productivity within ferruginous SCML may help to understand the environmental controls on primary productivity in past ferruginous oceans.

## Graphical abstract

This contribution details an investigation into the phytoplankton dynamics within a dimictic ferruginous lake in northern Germany. This lake has a pronounced subsurface chlorophyll maximum layer (SCML), and our work sought to explore whether this was a biomass or productivity maximum, and how the phytoplankton composition changes throughout the summer season. During late summer when the lake is maximally stratified, the SCML overlaps a redoxcline between dissolved iron and oxygen. The abundance of the micronutrient iron within the SCML in fall, but not in spring, offered an opportunity to explore how this micronutrient might impact the phytoplankton community structure and the productivity of the SCML.

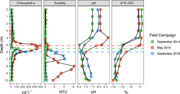

## 1 INTRODUCTION

The phenomenon of a subsurface chlorophyll maximum layer (SCML) describes a laterally extensive enrichment in the photosynthetic pigment Chlorophyll *a* in stratified marine and freshwater systems. SCML can co‐occur with biomass and/or a primary productivity maximum. Marine SCML are estimated to account for 47% of global net primary productivity (NPP) and can be a zone of new production versus recycled production that dominates in the mixed layer (Silsbe & Malkin, 2016). In stratified mesotrophic or eutrophic freshwaters, an SCML often forms at or below the thermocline depth where there is an optimum of light and/or nutrients (Leach et al., 2018). The position of the SCML can also be a dynamic motility response to diel fluctuations in light (Gervais, 1997), and its formation can be influenced by zooplankton grazing (Moeller et al., 2019). SCML can also be a photoacclimation response of phytoplankton where the amount of chlorophyll is enhanced without a change in biomass density. This usually occurs in oligotrophic waters where nutrients are most available at the base of the photic zone (Fennel & Boss, 2003).

Nutrient availability within an SCML is generally discussed in terms of nitrogen or phosphorus (i.e., N or P). But iron (Fe) is one of the most abundant metals in phytoplankton biomass (Ho et al., 2003), indicating that low environmental availability could also limit phytoplankton growth and/or primary productivity. It is a ubiquitous redox carrier in cytochromes, ferredoxin, and Fe–S proteins involved in electron transport during photosynthesis (Twining & Baines, 2013). Iron is the limiting nutrient in the high nitrate low chlorophyll (HNLC) regions of the ocean (Boyd et al., 2000), and a (co)‐limiting nutrient in some lakes (Havens et al., 2011; North et al., 2007; Vrede & Tranvik, 2006). Iron limitation has been described within marine SCML (Hogle et al., 2018), but iron bioavailability is generally not considered in investigations of SCML in lakes. This may be because iron is not frequently measured in lakes as it is not often found to be a limiting nutrient in lakes (Leung et al., 2021).

In oxygenated waters, the thermodynamically stable form of iron is Fe^3+^ (ferric), which is poorly soluble at circumneutral pH and rapidly precipitates as Fe^3+^ (oxyhydr)oxides. Removal of this mineralized iron to the sediments results in low iron bioavailability in the photic zone. While eukaryotic phytoplankton generally requires unchelated Fe^3+^, some cyanobacteria produce siderophores, which chelate Fe^3+^ and facilitate acquisition (Morrissey & Bowler, 2012). Due to its requirement as a nutrient by phytoplankton, the presence of ligand‐bound forms, and scavenging into a particle, iron generally displays a hybrid‐type element profile in marine systems (Bruland & Lohan, 2003). While many of the same processes control iron distribution in lakes, iron concentrations are generally higher in lakes than in oceans due to smaller basins and shorter water residence times that allow the iron to achieve higher concentrations. In lakes where thermal stratification gives rise to anoxic hypolimnia, sedimenting Fe^3+^ (oxyhydr)oxides can be reductively dissolved to form the much more soluble Fe^2+^ species. “Ferruginous” lakes are those with abundant dissolved iron, predominantly as Fe^2+^, in the hypolimnion enabled by seasonal or permanent stratification, which can result in a redoxcline between dissolved iron and oxygen. Although ferruginous conditions are generally not found in modern marine basins, they were a common feature of the deepwater of Precambrian oceans (Poulton & Canfield, 2011; Swanner et al., 2020).

SCML are common features of ferruginous lakes (Baker & Brook, 1971; Boehrer et al., 2017; Savvichev et al., 2017; Swanner et al., 2020), and can overlap with the dissolved iron‐oxygen redoxcline. The dominant phytoplankton within ferruginous SCML can be cyanobacteria (Baker & Brook, 1971; Savvichev et al., 2017), or diatoms (Swain, 1984), but this has also not been determined for many ferruginous lakes (Boehrer et al., 2017). Nevertheless, the abundance of dissolved iron and its predominantly ferrous form within the anoxic conditions of the hypolimnion has potential implications for phytoplankton composition and primary productivity in lakes. For instance, Cyanobacteria growth in an SCML overlapping a redoxcline in a ferruginous lake was linked to enhanced Fe^2+^ availability (Dillon & Molot, 2005; Molot et al., 2014). If enhanced availability of Fe^2+^ is associated with elevated biomass or primary productivity, it could have implications for estimates of primary productivity from lacustrine systems with anoxic hypolimnia. Lake hypoxia is potentially expanding due to land‐use changes and climate change (Jenny et al., 2016; North et al., 2014). As ferruginous conditions may be common in lake‐rich areas in northern boreal and deglaciated regions (Schiff et al., 2017; Swanner et al., 2020), the role of hypolimnetic dissolved iron on aquatic primary productivity in these regions is worth investigating.

The questions we address are which phytoplankton predominate within a ferruginous SCML, if their presence is related to the abundance of dissolved iron, and whether a ferruginous SCML is a locus of biomass and/or primary productivity. In this study, we pose these questions through an investigation of the geochemistry and phytoplankton community composition of a seasonally ferruginous dimictic lake with a persistent SCML, the Grosses Heiliges Meer (GHM). The SCML occurs within nonferruginous conditions in the spring but is colocated to the dissolved iron‐oxygen redoxcline under ferruginous conditions that develop in the fall, allowing for an investigation of the potential effects of dissolved iron on the SCML.

## 2 MATERIALS AND METHODS

### 2.1 Study site

**Figure 1:**
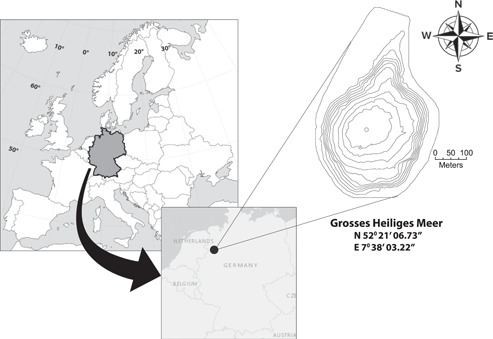
Map of Europe, highlighting Germany and the location of the GHM. Contour intervals are 1 m. GHM, Grosses Heiliges Meer.

The GHM is located near Hopsten, Germany at 52°21′06.73″ N and 7°38′03.22″ E (Figure 1). It has a surface area of 7.9 ha, a maximum depth of 10.8 m, and an average depth of 4.4 m based on a survey in 1993 by the Institut für Angewandte Ökologie und Gewässerkunde, Niederzier, Germany. It becomes thermally stratified from about mid‐April to late October. The GHM is part of an established field station in a protected area of the Landschaftsverband Westfalen‐Lippe (LWL) Museum für Naturkunde (Museum of Natural History). The GHM is one of several sinkhole lakes in the area, whose water chemistry varies as a function of the age of the lake, due to interaction with the groundwater of varying chemical properties (Pott, 2009). There are no natural surface water inlets or outflows, although until 1968 an industrial drainage ditch, the Meerbecke, flowed through the lake. This drainage has since been relocated to flow next to the lake. Iron in the lake is presumed to have been supplied directly by the Meerbecke in the past, or through the current Meerbecke‐groundwater system, as groundwater iron concentrations are highest in wells lying between the lake and the Meerbecke (Pust, 1993).

Samples were taken from a rowboat at a fixed mooring in the middle of the lake, with a water depth of approximately 10 m. There exists a record of physicochemical profiles that extend to when the field station was established in the 1930s (Chmiel, 2010; Terlutter, 2009; Table A1). Samples were collected in two fall time points: September 17, 2014, and September 13, 2018, and one spring timepoint: May 13, 2015. Although ideally, all sampling trips would have taken place in consecutive years, the long interval between sampling was due to investigators moving institutions.

### 2.2 On‐site measurements

Temperature and dissolved oxygen were measured with a Thermo Scientific Orion Star A Series optical oxygen and temperature sensor. The sensor had a 1‐point calibration with air‐saturated water. Conductivity was measured with a WTW Cond 170i meter connected to a TetraCon 325 electrode. Chlorophyll *a* was measured in situ using a Turner Designs SCUFA submersible fluorometer. Water clarity was determined using a Secchi disk.

### 2.3 Chemical analyses

Samples were pumped from depth to the surface using a groundwater pump. A WTW Multi 340i meter and Sentix pH electrode were used to measure pH on freshly retrieved samples. The electrode was calibrated with buffers of pH 4, 7, and 10. The CO_2_ concentration was determined by titration with 0.02 M NaOH to pH 8.2, using the phenolphthalein indicator. Where iron was present in the water column, these values are not reported, as precipitation of freshly oxidized iron consumes hydroxyl ion and interferes with this measurement.

Water samples were pumped from depth to the boat and immediately filtered using 0.45 µm cellulose nitrate filters. Samples for cations were acidified with five drops of nitric acid (65%) and stored at 4°C before analysis on an ICP‐OES. Samples for anions were stored at 4°C until analysis on an ion chromatograph (Metrohm 761 Compact Instrument). Anions and cations were measured at the University of Münster. Dissolved inorganic carbon (DIC) was measured on filtered samples stored with no headspace by titration with 0.1 M hydrochloric acid to pH 4.3, and δ^13^C‐DIC was measured on a GasBench interfaced to a Thermo Finnigan Delta Plus isotope ratio mass spectrometer (IRMS). Isotope results are reported as per mil (‰) difference to the VPDB standard.

### 2.4 Multi‐wavelength chlorophyll and photosynthetic measurements

In September 2018, samples were retrieved from discrete depths in the water column for taxon‐specific chlorophyll fluorescence and maximum quantum yield with a Walz Phyto‐PAM II (Heinz Walz GmbH). The Phyto‐PAM II is a multi‐wavelength fluorometer that deconvolutes fluorescence of Chlorophyll *a*, other chlorophylls, and/or accessory pigments at five wavelengths and quantitatively attributes the signal to one of four taxonomic groups: Cyanobacteria, diatoms, and dinoflagellates, Chlorophyta, and Cryptophya. The Phyto‐PAM II can additionally perform pulse‐amplitude modulate (PAM) fluorescence experiments, specifically by providing saturating pulses of light and deconvolute the taxon‐specific signals. After a period of >20 min. of dark adaptation, 3–4 ml samples were introduced into the instrument for the determination of chlorophyll concentration and maximum quantum yield (variable to maximum fluorescence; *F_v_*/*F_m_*) of photosystem II, or the photosynthetic efficiency (Cosgrove & Borowitzka, 2010). All samples were run in triplicate, and data are presented as the average of triplicates with two standard deviations. To remove background fluorescence attributed to dissolved organic carbon rather than photosynthetic pigments, water was filtered through 0.22 μm polyethersulfone (PES) filters, analyzed, and the results were subtracted from the raw water signals (i.e., a *Z*_off_ correction). Samples were agitated continuously with a magnetic stirrer during analysis. The Phyto‐PAM II has a detection limit of 0.1 μg L^−1^ chlorophyll. Taxon‐specific maximum quantum yields were either zero or had very high errors among triplicate measurements when chlorophyll concentrations for that taxon were <1 μg L^−1^. Therefore, maximum quantum yields were not reported when the corresponding taxon‐specific chlorophyll values for that sample were <1 μg L^−1^. The significance of maximum quantum yields between depths was assessed with a non‐parametric Kruskal–Wallis test, as not all taxa produced maximum quantum yields at all depths.

Similar measurements were made in September 2014 and May 2015 using a Water‐PAM (Heinz Walz GmbH). However, this instrument was only sensitive to fluorescence from Cyanobacteria. Also, measurements were made on samples returned to the laboratory more than 24 h after collection, and so are not appropriate to assess maximum quantum yields. In September 2018, we had the opportunity to make measurements on‐site for multiple taxa using the Phyto‐PAM, justifying the additional sampling campaign.

### 2.5 Most probable number (MPN)

MPN incubations were carried out in September 2014 and May 2015 to quantify active Cyanobacteria. The major salts of “Holy Medium” were modified from BG11 (Stanier et al., 1971) to better represent the major element chemistry of the GHM. The 100× stock solution of macronutrients contained 1 g NaNO_3_, 1.25 g MgSO_4_ × 7H_2_O, 2 g CaCl_2_ × 2H_2_O, and 0.05 g Na_2_‐EDTA in 500 ml of ultrapure water. No organic carbon sources were added to prevent heterotrophic growth, and cycloheximide was added at a concentration of 100 μg ml^−1^ to inhibit eukaryotic growth (Ferris & Hirsch, 1991). Dilution series were made in “Holy Medium” nongrowth buffer, which lacked trace metals, K_2_HPO_4_, ferric ammonium citrate, Na_2_CO_3_, and vitamins. For the MPN plates, 1% Noble agar was added to liquid media before autoclaving. Each well contained 0.9 ml of medium and 0.1 ml of diluted sample. Plates were incubated for 6–8 weeks at 20°C (depths of 0–5.5 m) and 10°C (depths 6–10 m). Wells were scored as positive growth based on turbidity, which was in most cases accompanied by visible green coloration. MPN and confidence intervals were calculated using MPNcalc v1.2.0 (https://mpncalc.galaxytrakr.org/).

### 2.6 DNA extraction and 16S and 18S ribosomal RNA (rRNA) gene amplicon sequencing

In May 2015, water was collected from every 1 m depth plus 5.1 m, the chlorophyll maximum detected with the submersible fluorometer. Between 75 and 100 ml was filtered onto 0.22 μm filters per depth. DNA was extracted with a DNEasy Powersoil DNA Isolation kit. The Powersoil kit is not generally applied to algae but it is effective for prokaryotic and eukaryotic DNA from aquaculture samples (Pearman et al., 2020). DNA was quantified with a Nanodrop. In September 2018, water was collected from every meter depth, plus 5.8 and 6.2 m, bracketing the 6 m chlorophyll maximum detected with the in situ probe. Water was filtered in series through 11 µm nylon and 0.22 μm PES filters to capture eukaryotic algae and bacteria (and archaea), respectively. DNA was extracted from filters using a DNeasy PowerBiofilm kit (Mäki et al., 2017), and DNA was quantified with a Qubit.

Illumina MiSeq was used to sequence the V4 region of the 16S rRNA gene with the primer pair 515 F (5′‐GTGCCAGCMGCCGCGGTAA‐3′) and 805R (5′‐GACTACVSGGGTATCTAAT‐3′) using a dual index approach (Gohl et al., 2016; Kozich et al., 2013). 16S rRNA gene amplicon sequencing was performed at the University of Minnesota Genomics Center on DNA extracts from 0.22 μm filters using 2 × 300 bp chemistry. 18S rRNA gene sequencing for eukaryotic algae was performed on an Illumina MiSeq using 2 × 250 bp chemistry at the Iowa State University DNA Facility on DNA extracts from 0.22 μm filters (May 2015) or 11 μm filters (September 2018). The V9 region 18S rRNA gene was amplified using primers 1391F (5′‐GTACACACCGCCCGTC‐3′) and EukBr (5′‐TGATCCTTCTGCAGGTTCACCTAC‐3′) (www.earthmicrobiome.org; Rii et al., 2018).

The 16S and 18S rRNA gene data were processed using the standard protocol in Mothur (version 1.39; Schloss et al., 2009). Out of 990,084 total 16S rRNA gene sequences, 745,127 sequences were retained after they were checked for quality (quality score greater than 25, error <1%, ambiguities [N] removed) and assembled. Any 16S rRNA gene sequences less than 299 bp or greater than 375 bp were removed and the sequences were aligned to the V4 region of the SILVA database (version 138; Quast et al., 2013). Overhangs were removed to ensure sequences overlapped in the same region. The final alignment was determined to be 658 columns wide. Next, chimeras were checked with VSEARCH version 2.13.3 (Rognes et al., 2016), and then preclustered up to two nucleotide differences between sequences. As such, 115,696 unique sequences were identified. Representative operational taxonomic units (OTU) for 16S rRNA gene sequences were classified (Bayesian classifier) to the SILVA database and clustered at 97% similarity with an 80% confidence threshold and rarefied to the lowest sequencing depth. The OTUs assigned to the phylum Cyanobacteria were blasted (NCBI blastn) to validate taxonomic assignment.

Similarly, 18S rRNA gene sequences were subjected to a processing method much like the 16S rRNA gene sequences, where 915,958 sequences were recovered from 1,067,170 total sequences after quality checks were applied. 18S rRNA gene sequences with less than 250 bp or more than 375 bp in length were removed. Sequences were aligned to the V9 region of interest (created by aligning *Saccharomyces cerevisiae*, downloaded from NCBI) to the SILVA database. The final alignment was determined to be 816 columns wide after overhangs and chimeras were removed (by VSEARCH) and this resulted in 35,231 unique sequences identified. The 18S rRNA gene sequences were taxonomically classified using a Bayesian classifier with the PR^2^ database (version 4.72; Guillou et al., 2013) and OTUs were clustered at 97% similarity and 80% confidence threshold. Two samples contained only a few hundred sequences; these were removed before rarefaction in Phyloseq (version 1.36.0; McMurdie & Holmes, 2013). All sequencing data was visualized using Phyloseq.

### 2.7 Calculations and statistical analysis

Secchi disk depths were converted to *K*_d_ (m^−1^), the light attenuation coefficient of photosynthetically active radiation (PAR), by dividing the depth by 1.7 m. The depth of 1% of surface PAR (*Z*_1%_) was estimated by dividing ln(100) by *K*_d_ (Idso & Gilbert, 1974; Poole & Atkins, 1929). The depth of the thermocline was calculated using rLakeAnalyzer (Winslow et al., 2019). Dissolved oxygen saturation was determined from measured dissolved oxygen, water temperature, and specific conductance values and published partition coefficients for oxygen in equilibrium with the atmosphere (Benson & Krause, 1980, 1984).

Data analysis and graphing utilized R v. 3.5.3 and the package tidyverse (Wickham, 2017). To determine for relationships between environmental variables to cyanobacterial 16S rRNA gene orders and photosynthetic eukaryotic 18S rRNA gene classes, Spearman correlation was performed using R packages hmisc (Harrell, 2020) and corrplot (Wei & Simko, 2017). Only environmental variables that had data for all depths were assessed. For this analysis, 16S and 18S rRNA gene abundances were normalized to the maximum abundances of that taxon from all depths at the date of sampling to ensure a normal distribution of values, and physicochemical data were normalized so that values ranged from 0 to 1.

## 3 RESULTS AND DISCUSSION

### 3.1 Seasonal chemical and biological trends

**Figure 2:**
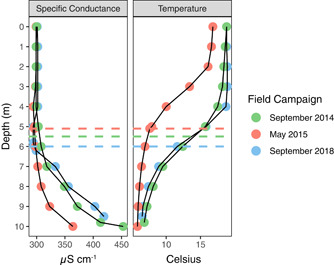
Physical measurements by depth in the GHM water column for the three field campaigns. The depth of the SCML for each sampling date is shown as dashed lines. GHM, Grosses Heiliges Meer; SCML, subsurface chlorophyll maxima layers.

**Figure 3:**
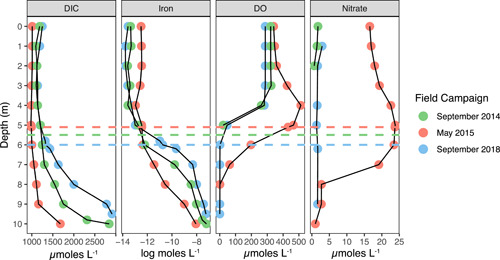
Measurements of the dissolved chemical constituents with depth in the GHM water column for the three field campaigns. DIC, dissolved inorganic carbon; DO, dissolved oxygen. The absence of data points for nitrate indicates values below the detection limit (see Methods). The depth of the SCML for each sampling date is shown as dashed lines. GHM, Grosses Heiliges Meer; SCML, subsurface chlorophyll maxima layers.

Physicochemical data (specific conductance and temperature) reveal both chemical and thermal stratification during the three sampling trips in September 2014, May 2015, and September 2018 (Figure 2). The depth of the thermocline was shallowest in May 2015 (2.9 m) compared to September 2014 (5.5 m) or September 2018 (5.2 m; Table 1). The euphotic depth (1% PAR) calculated from the Secchi disk readings was always below the thermocline and was shallowest in September 2014 (8.4 m) compared to May 2015 and September 2018 (9.2 m; Table 1). Chemical stratification, as evidenced by a gradient in specific conductance within the water column is most pronounced in fall, as both September dates have sharper chemoclines than May 2015, and the hypolimnion has a higher maximum specific conductance (Figure 2). Chemical changes throughout the water column are shown in Figure 3. The DIC profiles follow closely the specific conductance profiles. The magnitude of DIC was in the mM‐range, indicating that DIC is likely a major chemical constituent contributing to the increase in specific conductance with depth. Other major ions are likely to be Na^+^, Ca^2+^, K^+^, Cl^−^, and SO_4_^2−^, which are all abundant in local groundwater (Pust, 1993). During late summer stagnation, dissolved iron is typically present in the deep water column up to 5 m below the surface (Terlutter, 2009). A redoxcline of rapidly decreasing dissolved oxygen and increasing dissolved iron was always present in the GHM. The depth of this redoxcline was shallower in September 2014 and 2018 than in May 2015.

**Table 1:** Measured and calculated depths of limnological significance.

	Measured		Calculated		
	Depth of SCML (max. chl *a*)	Secchi depth	Depth of thermocline	1% PAR depth	Dissolved oxygen > air sat.
May 2015	5.1 m	3.40 m	2.9 m	9.2 m	0 to 6 m
September 2014	5.5 m	3.10 m	5.5 m	8.4 m	0 to 3 m
September 2018	6 m	3.40 m	5.2 m	9.2 m	None

Abbreviation: PAR, photosynthetically active radiation; SCML, subsurface chlorophyll maxima layers.

**Figure 4:**
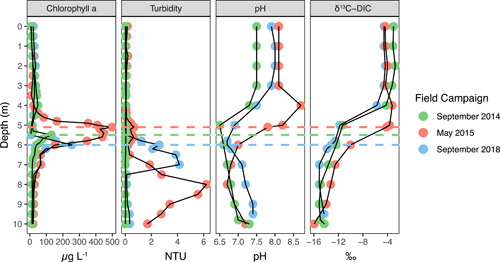
Measurements of biological parameters with depth in the GHM water column for the three field campaigns. Chlorophyll and turbidity were measured by probes, while pH and δ^13^C_DIC_ were measured on retrieved samples. The Secchi disk depths were 3.10 m in September 2014, and 3.40 m in both May 2015 and September 2018. The depth of the SCML for each sampling date is shown as dashed lines. GHM, Grosses Heiliges Meer; SCML, subsurface chlorophyll maxima layers.

Chlorophyll *a* as measured by the submersible fluorometer had a subsurface maximum in each field campaign, which varied in magnitude and depth. This SCM was the shallowest, widest, and maximum observed values in May 2015, reaching a maximum concentration of 498 μg L^−1^ at 5.1 m (Figure 4). The SCM was deeper and had a smaller magnitude in September 2014, with a maximum concentration of 130 μg L^−1^ at 5.5 m, and a maximum concentration of 254 μg L^−1^ occurred at 6 m in September 2018. Although profiles were not acquired across the lake basin to verify whether the SCM formed a continuous layer, monitoring from the mooring for a year detected an SCM from May to September (Table A2), suggesting it is a persistent feature during summer stratification that is likely to be laterally extensive. The term SCML is therefore applied. The shallowing of the redoxcline through the summer allowed the SCML to overlap with the redoxcline in fall (September 2014 and 2018), but the SCML and redoxcline were vertically separated in spring (May 2015).

**Figure 5:**
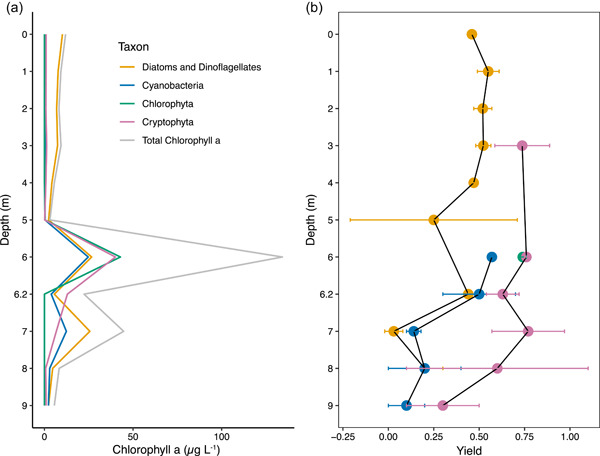
(a) The taxonomic distribution of chlorophyll *a* determined from the multi‐wavelength fluorometer in September 2018: Chlorophyta, Cryptophyta, Cyanobacteria, and Diatoms & Dinoflagellates. (b) Maximum quantum yield of Photosystem II (*F_v_*/*Fm*). The SCML was at 6 m in September 2018. There was no significant difference in maximum quantum yields for all taxa between depths as determined by a Kruskal–Wallis test. SCML, subsurface chlorophyll maxima layers.

Total chlorophyll abundance determined by in vivo multi‐wavelength fluorescence in September 2018 increased above antecedent levels in the epilimnion and hypolimnion at 6, 6.2, and 7 m (Figure 5), roughly matching trends observed with the submersible fluorometer (Figure 4). The chlorophyll concentrations determined by the submersible fluorometer are calibrated with Rhodamine WT, whereas the references used for calibration of the in vivo multi‐wavelength fluorometer were tied to laboratory‐determined chlorophyll concentrations quantified after extraction. Quantification in the submersible fluorometer can also be affected by temperature and the history of light exposure of the photosynthetic organisms, whereas samples were dark acclimated before in vivo multi‐wavelength fluorescence analysis to reduce these errors. Thus, the in vivo multi‐wavelength fluorescence measurements are likely more accurate for chlorophyll quantification. The submersible fluorometer data had higher spatial resolution and is presented because it gives the best sense of the structure of the water column.

### 3.2 Seasonal variation in GHM and SCML photosynthetic community

The data sets that address the question of which phytoplankton inhabit the SCML under ferruginous versus nonferruginous conditions are 16S and 18S rRNA gene sequencing. The limitations of this approach are that both 16S and 18S rDNA copy numbers in individual organisms can be highly variable, such that the abundance of a specific taxon (i.e., the fraction of reads belonging to a particular taxon in a sample) may not represent the actual proportions of one taxon's cellular abundance as compared to another. Sequencing also does not distinguish between live and dead organisms. In vivo multi‐wavelength chlorophyll fluorescence performed in September 2018 provides context as to which organisms were producing chlorophyll, as the amount of fluorescence attributable to different taxonomic groups is quantified by fitting the spectra with combinations of representative laboratory strain spectra. However, the multi‐wavelength instrument was not available during the 2014 and 2015 field campaigns.

18S rRNA gene sequencing showed that diatoms and dinoflagellates together had greater abundances in September 2018 relative to May 2015. Photosynthetic taxa of the diatom phylum Ochrophyta were the most abundant eukaryotic phytoplankton from 0 to 2 m and had their lowest abundance at 4 m in May 2015 and within the SCML in September 2018. Their abundance may not be directly proportional to greater biomass as there are high copy numbers of 18S rDNA within dinoflagellates (Figueroa et al., 2014; Medinger et al., 2010) and eukaryotes in general given their larger genome size (Prokopowich et al., 2003). However, in September 2018 diatoms and dinoflagellates were the dominant phytoplankton contributing to chlorophyll fluorescence in the epilimnion (Figure 5), providing another line of evidence for the abundance of these organisms in September 2018. They were also the predominant taxa at 7 m in May 2015, below the SCML but still within the photic zone depth calculated from Secchi disk readings (8.4 m; Table 1). The proportion of Ochrophyta and Dinophyta sequences at the SCML in September 2018 was small, but there was a peak of chlorophyll fluorescence from this group. The large increase in Chlorophyta sequences within the SCML as compared to all other depths led to the smaller proportion of all other taxa at this depth.

**Figure 6:**
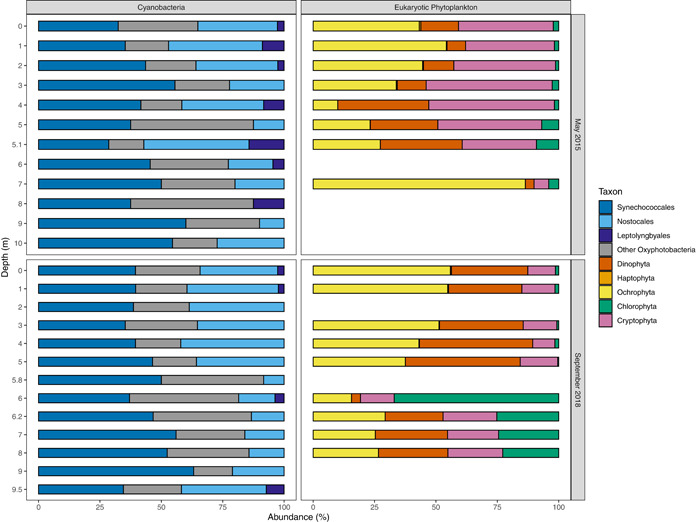
Proportions of Cyanobacteria and Eukaryotic Phytoplankton taxa from 16S and 18S rRNA gene amplicon sequencing data, in relation to the total reads of Cyanobacteria or Eukaryotic Phytoplankton. Haptophyta sequences were always <1% of 18S rRNA gene sequences. Cyanobacterial orders of low abundance (<0.1%) of all 16S rRNA gene reads in each sample have been removed. The SCML was at 5.1 m in May 2015 and 6 m in September 2018. rRNA, ribosomal RNA; SCML, subsurface chlorophyll maxima layers.

The Chlorophyta were of minor abundance in the 18S rRNA gene sequences in May 2015 but were the most abundant taxon recovered from the SCML in September 2018 (Figure 6). Most Chlorophyta sequences are derived from the classes Chlorophyceae and Trebouxiophyceae. The 18S rRNA gene copy number is highly variable across the Chlorophyta phylum, with one member of the Trebouxiophyceae having an average of 78.1 copies of the 18S rRNA gene (Gong & Marchetti, 2019). This suggests the enhanced abundance of Trebouxiophyceae 18S rRNA gene sequences within the SCML could be an artifact in September 2018. While microscopic cell counts would provide quantitative abundance on Chlorophyta, the multi‐wavelength chlorophyll data from September 2018 also detected enhanced chlorophyll attributed to Chlorophyta within the SCML as compared to other depths (Figure 5). Chlorophyta 18S rRNA gene sequences from the September 2018 SCML are dominated by Trebuxiophyceae, whereas Chlorophyceae predominate in the epilimnion in May 2015, suggesting the community is actively turning over on seasonal timescales.

The existence of an SCML identified by chlorophyll abundance can indicate an enhancement of photosynthetic cells or an enhancement of chlorophyll within photosynthetic organisms. Enhancement of chlorophyll within organisms can occur independently from increases in phytoplankton biomass and can be an adaptation to low light conditions (Fennel & Boss, 2003). The photic zone depth was 9.2 m in September 2018 (Table 1). The SCML is centered at a depth below where optimal light conditions exist (Figure 4), 1–2 m below the Secchi disk depth but well within the calculated photic zone. It is, therefore, possible that Chlorophyta in the SCML have increased chlorophyll to adapt to low light. There is also a possibility that the Chlorophyta living below the optimal photic depth could utilize anaerobic or fermentative metabolisms within the anoxic SCML, such as are known from sequenced Chlorophyta genomes (Atteia et al., 2013). Such a lifestyle was suggested for Chlorophyta 18S rRNA gene sequences recovered from the aphotic monimolimnion of meromictic and ferruginous Lake Pavin (Lepère et al., 2016).

**Figure 7:**
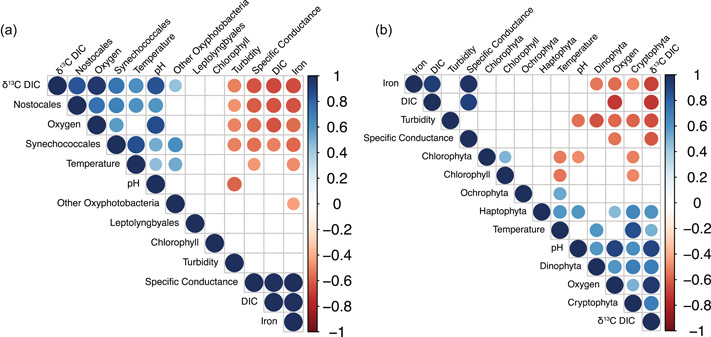
Spearman correlations calculated for environmental and the response variables. (a) 16S rRNA gene normalized abundance of cyanobacterial orders. (b) 18S rRNA gene normalized abundance of photosynthetic eukaryotic phyla for data from May 2015 and September 2018. Only those correlations that were significant (i.e., *p* < 0.05) are denoted with colored circles. Blue is a positive correlation and red is a negative correlation. rRNA, ribosomal RNA.

Cryptophyta constituted a greater proportion of the eukaryotic phytoplankton sequences in May 2015 than in September 2018. They were better represented in the epilimnion in May 2015, and below the redoxcline in September 2018. They constituted a considerable proportion of chlorophyll at the SCML in September 2018, although their sequencing proportion did not increase (Figure 5). Cryptophyta 18S rRNA gene abundance is positively correlated with oxygen and temperature (Figure 7), suggesting a preference for the epilimnion.

The combination of lower proportions of 18S rRNA gene sequencing and elevated chlorophyll fluorescence within the SCML suggests that Cryptophyta may adapt to the low light conditions of the SCML with enhanced pigment production. For instance, Cryptophyta synthesize phycoerythrin to harvest deeper‐penetrating blue light (MacColl, 1998). Another possibility to reconcile these observations is the misattribution of chlorophyll fluorescence to Cyptophyta when phycoerythrin‐containing Cyanobacteria are present (Catherine et al., 2012). Cryptophyta sequences were detected in 18S rRNA gene sequencing within the SCML, indicating that not all Cryptophyta fluorescence can be attributed to Cyanobacteria, it is possible that enhanced phycoerythrin production by low‐light adapted Cyanobacteria within the SCML gave rise to elevated fluorescence attributed to Cryptophya. Synechococcaceae dominate the Cyanobacterial sequences, and freshwater *Synechococcus* are known to synthesize phycoerythrin (Sánchez‐Baracaldo et al., 2019).

Oxygenic cyanobacterial 16S rRNA gene sequences were detectable at all depths sampled (Figure 6). Sequences represented the following orders, with the average 16S rRNA gene copy number and standard deviation of that order in parenthesis, where known (Table A3): Synechococcales (2.0 ± 0.7), Nostocales (4.0 ± 0.9), Chroococcales (2.3 ± 0.5), Oscillatoriales (3.0 ± 1.0), and Pleuocapsales, listed in order of decreasing abundance in the data set. Numerous unclassified cyanobacterial sequences were also recovered. Sequences were also recovered from the candidate phylum Melainabacteria, a sister phylum of Cyanobacteria that contains organisms that are likely to be non‐photosynthetic (Soo et al., 2017). These organisms were most abundant in the SCML in May 2015 and within the hypolimnion in September 2018. One of the only cultured representatives of this candidate phylum is *Vampirovibrio chlorellavorus*, a predator of Chlorophyta (Soo et al., 2017). Chlorophyll from Chlorophyta was most abundant within the SCML in September 2018. Cyanobacteria dominated chlorophyll fluorescence in the hypolimnion. Cyanobacteria are known to synthesize accessory pigments such as phycoerythrin to help harvest light of different wavelengths, particularly shorter wavelengths that preferentially penetrate through the water column or benthos (Hogle et al., 2018), which could have contributed to their increase in fluorescence.

### 3.3 Relationship of iron to organisms

The seasonal variation in iron abundance within the SCML is useful to pose the question of whether the abundance of dissolved iron has any effect on the identity of the phytoplankton within the ferruginous SCML. A limitation of this system for answering the question of whether iron abundance influenced the phytoplankton community is that other nutrients and physicochemical conditions also vary seasonally.

Dissolved iron was 22 μM at 6 m in September 2018, but ~1 μM in the epilimnion, raising the possibility that enhanced iron availability was related to enhanced Chlorophyta within the SCML in September 2018 in both the 16S rRNA gene and multi‐wavelength chlorophyll fluorescence datasets. Chlorophyta have a higher iron demand than other eukaryotic phytoplankton (Ho et al., 2003). However, there was no correlation between Chlorophyta 18S rRNA gene relative abundance and dissolved iron concentrations (Figure 7). The sequential filter sizes used for DNA extraction in September 2018 helped avoid filter clogging and avoid bias in amplification due to the abundance of large organisms with high 18S rRNA gene copy numbers (Muñoz‐Colmenero et al., 2021). However, some smaller eukaryotic algae, including some Chlorophyta, are not captured on larger pore size filters and could have been missed in September 2018 18S rRNA gene sequencing. Nitrate was in low abundance throughout the water column and phosphate was not detected within the SCML in September 2018 as compared to May 2015. It, therefore, seems unlikely that nitrogen or phosphorus availability stimulated Chlorophyta in the September 2018 SCML. Further work would be required to support or reject the hypothesis that Chlorophyta are stimulated by enhanced iron availability in the SCML. Such work might include incubations where iron availability is manipulated and changes in Chlorophyta growth, activity, or intracellular iron concentrations are assessed.

Greater iron concentrations may also favor Cyanobacteria, as Fe:P ratios measured in phytoplankton are generally highest for Cyanobacteria, then Chlorophyta, then red and brown algae (Quigg et al., 2011). Prior research has also indicated that ferrous iron, which was likely a significant component of dissolved iron in the redoxcline in September 2018 given the low‐oxygen conditions (Figure 3), can preferentially stimulate the growth of Cyanobacteria relative to other phytoplankton (Molot et al., 2014). The order *Synechococcales* had a local maximum at the SCML and dominated the Cyanobacteria at that depth. The coastal cyanobacteria *Synechococcus* have higher iron quotas and high Fe:C ratios as compared to eukaryotic phytoplankton under low‐light conditions (Sunda & Huntsman, 2015). Nevertheless, there was no significant correlation between any Cyanobacteria 18 S rRNA gene taxa and dissolved iron (Figure 7). A lack of correlation could be an effect of the limited number of samples (*n* = 21), or could arise from a complex response to multiple environmental variables that vary with depth (e.g., light, nitrogen, phosphorus, and iron availability). Diazotrophic Cyanobacteria also have enhanced iron requirements as compared to non‐diazotrophs (Tortell et al., 1999). However, the diazotrophic order *Nostococcales* was more abundant in the epilimnion in September 2018 relative to May 2015, when nitrate was much less abundant (Figure 3), despite lower dissolved iron concentrations at that time, suggesting a response to nitrogen limitation rather than iron availability.

### 3.4 Enhanced biomass within the SCML?

SCML can be zones of enhanced biomass or just enhanced chlorophyll. Cell counts could have discriminated between these possibilities for the GHM if organisms were identified and biovolume accounted for. While cell counts were performed in September 2014 and May 2015, only total cells were quantified, not biovolume (see Appendix). It is therefore not possible to determine if the enhancement of cells below SCML in September 2014 is driven by photosynthetic organisms, or bacteria, which are also abundant within the chemoclines of stratified lakes (Savvichev et al., 2017).

The MPN results provide context as to the quantities of viable, cultivatable Cyanobacteria, which can give some semi‐quantitative context to the relative abundances of Cyanobacteria determined from the 16S rRNA gene data set. It should be noted that MPN was performed on September 2014 samples, while 16S rRNA gene sequencing was performed on September 2018 samples, such that these numbers for fall cannot be directly compared. However, data collected at similar times of year but in different years show similar geochemical and chlorophyll profiles (Tables A1 and A2).

**Figure 8:**
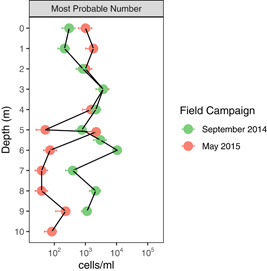
MPN for cyanobacteria in September 2014 and May 2015. Error bars represent 95% confidence intervals as log values */the abundances. The SCML was at 5.5 m in September 2014 and 5.1 m in May 2015. MPN, most probable number; SCML, subsurface chlorophyll maxima layers.

The MPN results show generally more abundant culturable Cyanobacteria at 0 and 1 m in May 2015 than in September 2014, and more abundant culturable Cyanobacteria below 5 m in September 2014 than in May 2015 (Figure 8). At both sampling times, there is a subsurface peak in MPN Cyanobacteria that co‐occurred with the SCML at 5.1 m in May 2015 but was at 6 m in September 2014, just below the SCML at 5.5 m. These could indicate that the GHM SCML does have elevated cyanobacterial biomass, although the maximum of MPN Cyanobacteria appears to be below the SCML in the fall, but also co‐occurs with a maximum of counted cells (see Appendix). This suggests the bottom of the SCML could be a zone of increased biomass for Cyanobacteria in the fall, under ferruginous conditions. The ability to adapt to low light conditions might also help to explain why cyanobacterial 16S rRNA gene sequences are detected in low light conditions at the bottom of SCML and in the hypolimnion. The observation based on in vivo multi‐wavelength chlorophyll measurements in September 2018 that Cyanobacteria dominated chlorophyll in the hypolimnion, as well as the detection of cultivatable Cyanobacteria via MPN, indicate that Cyanobacteria are still active at these depths, despite likely light limitation. The hypothesis that the SCML results from an enhancement of chlorophyll but not biomass does not seem to be supported for Cyanobacteria in the GHM. More detailed cell counts and biovolume estimates would help to discern whether the SCML has increased biomass.

### 3.5 Seasonal indicators of primary productivity

Another hypothesis regarding the origin of SCMLs is that they are biomass maxima that result from optimal conditions for primary productivity. Optimal conditions could include light, temperature, or nutrient availability. If the SCML were a primary productivity maximum, a prediction would be that indicators of primary productivity should co‐occur with the chlorophyll maximum. Neither gross nor net primary productivity was measured in this study, but several chemical species were assessed that are expected to vary because of photosynthesis and/or carbon fixation. These include oxygen, pH, and DIC/δ^13^C_DIC._ In addition, the maximum quantum yield determined on samples in September 2018 indicates a capacity for photosynthesis and carbon fixation.

Oxygen can be introduced to the epilimnion from the atmosphere or produced in situ via oxygenic photosynthesis. If dissolved oxygen exceeds the value predicted to be due to air saturation, it indicates production that is outpacing consumption due to respiration. In May 2015, the lake had a deep oxygen maximum centered around 4 m (145% air saturation; Figure 3), and dissolved oxygen was above air saturation from 0 to 5.1 m (SCML; Table 1). Dissolved oxygen and pH were correlated throughout the water column, and elevated pH also occurred at 4 m depth in May 2015 (Figure 7). Elevated pH can result during the growth of phytoplankton due to uptake of bicarbonate (Schultze‐Lam et al., 1997), and is an indicator of carbon fixation in phytoplankton.

Trends in DIC and δ^13^C_DIC_ in aquatic systems can reflect carbon fixation, but they also incorporate signals from processes such as respiration and chemical equilibration (i.e., CO_2_ dissolution/exsolution, (bi)carbonate (de)protonation, and mineral precipitation). In the GHM, DIC concentrations increased below the oxycline, as might be expected for photosynthetic carbon fixation in the euphotic zone and remineralization of DIC from exported organic carbon (Figure 3). In May 2015, a DIC minimum occurred at 5 m. The CO_2_ concentrations determined by titration had a minimum at 4 m in May 2015 (Swanner & Leung, 2021). In general, the δ^13^C_DIC_ was consistently lighter in the bottom 4 m of the GHM at all sampling times (−12 to −16‰), and heavier in the upper 4 m of the water column (−2 to −5‰). In May 2015 there was a δ^13^C_DIC_ excursion at 4 m to the heaviest values observed on that date (Figure 4). The shift to heavier δ^13^C_DIC_ from surrounding antecedent conditions can result from biological carbon fixation, which preferentially consumes ^12^C and leaves the residual DIC pool enriched in ^13^C. DIC and δ^13^C_DIC_ were anticorrelated, while δ^13^C_DIC_ was correlated with pH, and dissolved oxygen (Figure 7). Chemical interactions or dependencies of these environmental variables are not expected, other than through photosynthetic carbon fixation. Although particulate organic carbon (POC) quantities and δ^13^C_POC_ data (which were not collected) would be necessary to quantify primary productivity, the chemical data are consistent with elevated photosynthetic primary productivity at 4 m in May 2015, above the SCML at 5 m.

Dissolved oxygen was never above air saturation in September 2018 but exceeded air saturation from 0 to 3 m in September 2014 (Table 1). However, peak values were 110%, far less than peak values in May 2015. There was no excursion to elevated pH values in either September data set. A slight excursion to heavier δ^13^C_DIC_ occurred at the SCML in September 2018. Chemical indicators of primary productivity were more muted in September as compared to May, which may result from the lowered availability of nutrients in the epilimnion in September. This is evident for nitrate but also dissolved iron (Figure 3). The productivity indicators are also not as localized to a certain depth in September 2018 as in May 2015. For instance, the oxygen maximum occurs in the epilimnion in September 2014, while the heavy δ^13^C_DIC_ excursion occurs at 6 m in September 2018. This may suggest that the locus of primary productivity in the fall varies from year to year, or that other biogeochemical reactions co‐occurring at certain depths in fall could obscure these signals. For instance, vigorous respiration could consume oxygen. Oxygen could also be consumed by the oxidation of methane that is present in the hypolimnion but in low abundance in the epilimnion, which would produce isotopically light DIC at the redoxcline that could obfuscate a positive excursion resulting from carbon fixation. Wind‐driven mixing of the epilimnion could also disrupt these trends.

The maximum quantum yield measurements conducted in September 2018 give some insight into the capacity of each taxon for photosynthesis and carbon fixation at specific depths. Diatoms and dinoflagellates dominated the chlorophyll composition in the epilimnion and were the only taxa to consistently produce maximum quantum yield data from these depths. The maximum quantum yield for diatoms and dinoflagellates was in the epilimnion, and a maximum quantum yield was not detected for these organisms at the SCML (6 m), despite exceeding 1 μg L^−1^ chlorophyll (Figure 5). Lowered maximum quantum yield can indicate nutrient stress (Campbell et al., 1998), and nitrate preferentially stimulates diatoms (i.e., Dinophyta) in cold water in spring (Glibert et al., 2016). Nitrogen and phosphorus were more abundant in the epilimnion than the SCML in September 2018, indicating these organisms may have been nitrate‐limited within the September 2018 SCML. The much greater availability of nitrate in the epilimnion in May 2015 compared to the fall is likely a product of spring overturn.

The maximum quantum yield values for Cyanobacteria were highest within the SCML, dropped to near 0.1 in the hypolimnion, and were not detected in the epilimnion. The maximum quantum yield values for Cyanobacteria were low compared to the other photosynthetic taxa, which could result from their smaller size (Suggett et al., 2009). Elevated cultivatable Cyanobacteria were detected within the SCML in September 2014 with MPN counts. The fall SCML in the GHM had very low concentrations of nitrate (Figure 3), however, ammonium might be a nitrogen source within the redoxcline. Although ammonium was not routinely measured, we did attempt to measure ammonium with flow injection analysis on samples collected in May 2015. Ammonium was detected and showed a general trend of increasing abundance in the hypolimnion. However, quantification was not reliable, likely due to matrix interferences, and so these values are not reported. Elevated hypolimnetic ammonium concentrations have been noted below the oxycline of other ferruginous lakes (Lambrecht et al., 2018; Michiels et al., 2017), and are an expected product of anaerobic remineralization. Past lake studies have observed that Cyanobacteria dominate lakes later in the season when ammonium is more available (Glibert et al., 2016). The MPN and maximum quantum yield results indicate that Cyanobacteria could be more abundant and/or have enhanced photosynthetic capacity within the SCML and chemocline during the fall and suggests this observation should be further explored.

The maximum quantum yield measurements indicate that Chlorophyta are photosynthetically active in the SCML. Chlorophyta also had elevated 18S rRNA gene relative abundances and chlorophyll fluorescence within the SCML. The maximum quantum yield of 0.74 ± 0.02 measured for Chlorophyta at 6 m in September 2018 (Figure 5) is near the theoretical maximum quantum yield of 0.8 (Magnusson, 1997). Due to the absence or low abundance of Chlorophyta chlorophyll elsewhere in the water column, no other yield measurements were recorded for the taxon. This high maximum quantum yield may indicate that nutrient and/or light availability was optimal for Chlorophyta within the SCML (Rattan et al., 2012).

Cryptophyta had the highest maximum quantum yield values of all taxa. Cryptophyta, along with Haptophyta and *Synechococcales*, had positive correlations between their 16S or 18S rRNA gene relative abundance and dissolved oxygen, pH, and δ^13^C_DIC_ (Figure 7). The 18 S rRNA gene sequences of these organisms were the most abundant of all 18S rRNA gene oxygenic photosynthetic sequences at 4 m in May 2015. These observations support the Cryptophyta as potentially important taxa for primary productivity in the GHM.

### 3.6 Insights into phytoplankton dynamics in ferruginous oceans

Ferruginous lakes such as the GHM have become important analogs to past ferruginous oceans because the modern oceans are generally oxygenated and have extremely low concentrations of iron. While the physical factors driving mixing are very different between lakes and oceans, some of the chemical and biological features of modern ferruginous lakes, such as their lower sulfate content, can approximate aspects of past ferruginous oceans (Swanner et al., 2020). This study provides data on the distribution of phytoplankton and indicators of primary productivity and gives some insight into what factors might be important to the relationship between the SCML and primary production in ferruginous conditions.

In this study, indicators of biomass and productivity maxima were spatially separated from the SCML under non‐ferruginous conditions, but under ferruginous conditions, these coincided with the SCML. The phytoplankton with the highest iron demands, Cyanobacteria and Chlorophyta, had elevated abundance within the SCML, suggesting a ferruginous SCML could alleviate iron demands, although this was not supported by simple correlations, likely because reverse gradients of other required resources, such as light. It has also been proposed oxygen production would have been limited by the toxicity of reactive oxygen species (ROS) produced in the presence of Fe^2+^ in ferruginous oceans (Swanner et al., 2015). However, experiments investigating this phenomenon are generally carried out in monoculture under high‐light conditions (Herrmann et al., 2021). It is possible that the low‐light conditions likely for an SCML could help to diminish the production of ROS. Another possibility is that ROS‐mitigating bacteria within a redoxcline also help to decrease oxidative stress (Szeinbaum et al., 2021). Exploring these questions within ferruginous lakes should provide snapshots into how photoautotrophic processes are regulated by the different phytoplankton and microbial populations, as well as chemical and light conditions across a range of ferruginous lakes.

Much of the investigation into the ecology of primary producers in ferruginous lakes as analogs to past ferruginous oceans has focused on anoxygenic photosynthetic bacteria, particularly those that can use Fe^2+^ as a photosynthetic electron donor (Crowe et al., 2008; Llirós et al., 2015; Walter et al., 2014). This type of primary productivity is commonly invoked for past ferruginous oceans (Johnston et al., 2009; Kappler et al., 2005). Despite the potential for Fe^2+^‐based anoxygenic photosynthesis to fix significant carbon within portions of modern ferruginous lakes (Morana et al., 2016), there is generally still abundant oxygenic phytoplankton within these systems if the light is available (Savvichev et al., 2017; Swanner et al., 2020). Thus, oxygenic photosynthesis could still be a major photoautotrophic pathway within ferruginous water columns past and present, especially as anoxygenic photosynthetic bacteria can be confined to discrete layers dictated by electron donor and light availability. Significant chlorophyll, turbidity, and/or biomass maxima attributable to phytoplankton occur in ferruginous lakes, sometimes corresponding to the depth of the redoxcline (Baker & Brook, 1971; Swanner et al., 2020). Elucidating whether these layers are hot spots for primary productivity, whether oxygenic photosynthesis predominates over anoxygenic photosynthesis in these layers, and what environmental or ecological factors allow anoxygenic photosynthesis to flourish should be goals of future research into the ecology of ferruginous systems.

## 4 CONCLUSIONS

The SCML is a seasonally persistent feature of the GHM, a dimictic ferruginous lake. However, the biological and geochemical characteristics of the SCML vary strongly between the spring and fall seasons. In the spring, the SCML does not appear to be a zone of enhanced primary productivity. Several indicators of enhanced primary productivity, including dissolved oxygen, pH, and a heavier δ^13^C_DIC_ excursion occur at a shallower depth than the SCML. In the fall, the magnitude of the SCML diminishes, but it co‐occurs with a redoxcline between dissolved iron and oxygen. There are enhanced cultivatable Cyanobacteria that the SCML in the fall when dissolved iron was elevated. There is also a slight positive δ^13^C_DIC_ excursion within the SCML, and the highest maximum quantum yields Cyanobacteria, and only detected maximum quantum yield for Chlorophyta, indicating the potential for enhanced photosynthesis and carbon fixation at this depth. Although Cyanobacteria have higher iron demands than other phytoplankton, and Chlorophyta have higher demands than other eukaryotic phytoplankton, there was no statistically significant relationship between dissolved iron and Cyanobacteria or green algal abundance indicators. This study provides insights into questions about how dissolved iron might influence primary productivity in ferruginous lakes. As complex bottom‐up and top‐down factors give rise to SCML in aquatic systems (Cullen, 2015), investigating the relationship between dissolved iron and phytoplankton and photosynthesis in a range of ferruginous systems can help to identify the environmental triggers for the development and maintenance of a ferruginous SCML, as well as factors influencing primary productivity and oxygen production. With further study, such insights might provide context for the locus and mode of primary productivity in past ferruginous oceans.

## AUTHOR CONTRIBUTIONS

**Elizabeth D. Swanner**: conceptualization (lead); data curation (lead); formal analysis (equal); funding acquisition (lead); investigation (lead); methodology (lead); project administration (lead); supervision (lead); visualization (equal); writing – original draft (lead); formal analysis (lead); writing – review and editing (lead); **Marina Wüstner**: investigation (supporting); methodology (supporting); writing – review and editing (supporting); **Tania Leung**: data curation (supporting); formal analysis (equal); visualization (equal); writing – review and editing (supporting); **Jürgen Pust**: investigation (supporting); methodology (supporting); writing – review and editing (supporting); **Micah Fatka**: formal analysis (supporting); writing – review and editing (supporting); **Nick Lambrecht**: formal analysis (supporting); writing – review and editing (supporting); **Hannah E. Chmiel**: investigation (supporting); methodology (supporting); writing – review and editing (supporting); **Harald Strauss**: investigation (supporting); methodology (supporting); writing – review and editing (supporting).

## CONFLICTS OF INTEREST

The authors declare no conflicts of interest.

## ETHICS STATEMENT

None required.

## Figures and Tables

**Figure A1:**
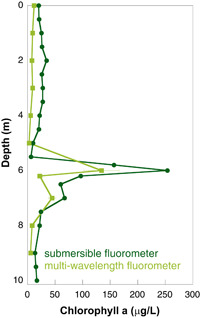
Multiple measurements of chlorophyll with depth in the GHM water column in September 2018. The submersible fluorometer produced systematically higher chlorophyll concentrations than the multi‐wavelength fluorometer. GHM, Grosses Heiliges Meer.

**Figure A2:**
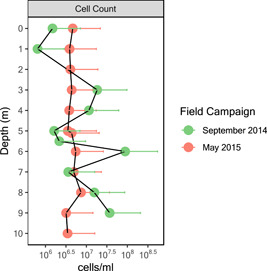
Cell counts in September 2014 and May 2015. Error bars represent 95% confidence intervals as log values */the abundances.

## Data Availability

All sequencing data are available in the NCBI repository under BioProject PRJNA720690: https://www.ncbi.nlm.nih.gov/bioproject/PRJNA720690. Physical, chemical, and biological data utilized in this study are available in figshare at https://doi.org/10.25380/iastate.14394455.v1.

## 1

The multi‐wavelength fluorescence observations documented the SCML, although the magnitude and depth of the maximum were systematically lower than from the in situ fluorometer (Figure A1). Light exposure upon collection may have quenched fluorescence in the samples analyzed by the multi‐wavelength fluorometer. These samples were dark acclimated for at least 20 min before measurement which should have minimized this effect (Huot & Babin, 2010). Fluorescence from organic matter might also produce higher chlorophyll a reading in the in situ fluorometer. However, the *Z*_off_ correction applied by analyzing filtered samples in the multi‐wavelength fluorometer was smaller than the variation between the two analyses, suggesting this interference had a minor effect. The differences in chlorophyll values most likely arise from the different calibrations between the two fluorometers. No attempt was made to validate the calibrations here, that is, with chlorophyll extractions. The in situ fluorometer is expected to be internally consistent and is used to compare trends in absolute quantities of chlorophyll from different sampling times and depths, while the multi‐wavelength fluorometer data are used to make comparisons between sampling depths in September 2018 only (Figure A2).

Select geochemical (Table A1) and submersible fluorometer chlorophyll (Table A2) data collected in 2010 by Chmiel (2010). These data show the development of seasonal stratification (i.e., dimixis) and the persistence of an SCML in the GHM from May to September. BDL = below detection limit; ND = not determined.

**Table A1:** Historical geochemical data from GHM.

Depth (m)	Temperature (**°**C)	pH	Specific conductance (μS/cm)	Dissolved oxygen (mg/L)	Dissolved iron (mg/L)
3/16/10	12:40	Secchi depth	1.6 m		
0	4.5	6.8	306	8.8	1.1
1	4.2	6.8	306	8.7	1.2
2	4.1	6.8	306	8.5	1.2
3	4.1	6.8	306	8.5	1.2
4	4.1	6.8	306	8.6	1.2
5	4.1	6.9	306	8.5	1.2
6	4.1	6.9	306	8.5	1.2
7	4.1	6.9	306	8.5	1.2
8	4.1	6.9	306	8.5	1.2
9	4.2	6.8	308	7.8	1.5
9.5	4.2	6.8	308	7.3	1.6
9.8	ND	6.6	340	1.1	10.1
10	4.4	6.7	370	BDL	18.2
5/5/10	14:20	Secchi depth	3.1 m		
0	14.0	7.9	303	11.8	0.1
1	13.9	7.9	303	11.6	0.1
2	13.3	7.9	303	11.7	0.1
3	11.8	7.9	300	14.0	0.1
4	7.6	6.9	301	11.3	0.1
5	5.7	6.8	304	7.8	0.1
6	5.0	6.6	307	3.2	0.3
7	4.7	6.6	311	0.7	0.3
7.5	4.8	6.5	312	BDL	0.4
8	4.7	6.6	308	BDL	0.9
8.5	4.7	6.6	320	BDL	1.3
9	4.7	6.7	330	BDL	1.6
9.5	4.8	6.8	350	BDL	6.4
10	4.8	7.0	374	BDL	13.1
8/10/10	16:00	Secchi depth	2.4 m		
0	22.5	8.1	307	10.3	ND
0.5	22.5	8.1	307	10.2	ND
1	22.0	8.2	306	10.5	ND
1.5	21.6	8.2	307	10.6	ND
2	21.4	8.2	307	10.6	ND
2.5	21.2	7.9	307	9.8	ND
3	20.9	7.7	307	9.6	ND
3.5	20.3	7.3	306	9.4	ND
4	18.1	6.9	302	8.9	ND
4.5	15.4	6.7	300	6.5	ND
5	13.0	6.5	302	2.9	ND
5.5	11.5	6.5	304	1.6	ND
6	9.7	6.5	307	0.3	ND
6.5	8.7	6.6	311	BDL	ND
7	7.5	6.6	321	BDL	ND
7.5	7.0	6.8	341	BDL	ND
8/11/10	13:00	Secchi depth	2.6 m		
0	22.1	8.0	306	10.3	ND
0.5	21.8	8.0	307	10.4	ND
1	21.8	8.0	306	10.4	ND
1.5	21.6	8.0	307	10.3	ND
2	21.6	8.0	307	10.2	ND
2.5	21.4	7.9	307	10.1	ND
3	21.0	7.5	307	9.4	ND
3.5	20.0	7.2	305	9.7	ND
4	18.1	6.8	302	8.7	ND
4.5	16.1	6.6	300	7.1	ND
5	13.7	6.4	301	3.9	ND
5.5	11.3	6.4	304	1.6	ND
6	9.8	6.4	308	0.3	ND
6.5	8.7	6.4	310	0.2	ND
7	7.7	6.5	308	BDL	ND
7.5	6.9	6.6	341	BDL	ND
9/13/10	10:50	Secchi depth	1.3 m		
0	16.9	7.2	285	10.6	0.4
1	16.5	7.2	285	10.2	0.3
2	16.4	7.0	285	8.5	0.4
3	16.2	6.9	285	7.3	0.3
4	16.0	6.7	288	6.1	0.7
5	15.1	6.6	293	1.4	0.6
6	11.9	6.6	312	0.5	3.5
7	8.9	6.8	332	BDL	7.7
8	7.3	7.0	364	BDL	15.5
9	6.5	7.1	415	BDL	27.8
9.9	6.2	7.3	465	BDL	41.2

Abbreviation: GHM, Grosses Heiliges Meer.

**Table A2:** Historical chlorophyll data from GHM.

Depth (m)	12/8/09 14:20	3/16/10 12:40	5/5/10 14:20	8/10/10 16:00	8/11/10 13:00
0	6.0	7.3	4.9	8.4	9.4
0.5	ND	11.6	5.9	12.7	10.2
1	6.0	10.5	5.9	20.7	13.7
1.5	ND	13.8	6.4	26.7	17.7
2	5.0	13.2	9.7	33.2	24.1
2.5	ND	13.3	10.0	36.4	24.4
3	5.0	13.3	15.2	31.5	26.5
3.5	ND	12.1	39.9	36.6	31.1
3.8	ND	ND	37.0	ND	ND
4	5.0	12.8	53.5	38.0	24.0
4.3	ND	ND	74.1	ND	ND
4.5	ND	12.4	53.8	47.0	28.3
4.8	ND	ND	37.0	ND	ND
5	5.0	12.0	35.7	22.8	15.6
5.5	ND	13.2	29.8	15.6	11.8
6	5.0	12.4	27.3	30.4	41.4
6.1	ND	ND	ND	54.0	27.7
6.2	ND	ND	ND	60.3	35.8
6.3	ND	ND	ND	60.5	51.2
6.4	ND	ND	ND	59.8	53.8
6.5	ND	12.1	19.3	31.1	94.9
6.6	ND	ND	ND	115.1	71.8
6.7	ND	ND	ND	211.0	187.4
6.8	ND	ND	ND	66.9	173.3
6.9	ND	ND	ND	50.0	122.3
7	5.0	11.9	19.1	52.3	44.3
7.2	ND	ND	ND	54.3	44.3
7.4	ND	ND	ND	56.8	49.6
7.5	ND	ND	17.3	58.7	ND
7.6	ND	ND	ND	60.3	51.9
7.7	ND	ND	ND	61.1	ND
7.8	ND	ND	ND	57.9	51.4
7.9	ND	ND	ND	52.7	ND
8	4.0	11.7	16.3	50.8	40.3
8.2	ND	ND	ND	40.4	ND
8.4	ND	ND	ND	39.3	ND
8.5	ND	12.1	11.7	ND	35.8
8.6	ND	ND	ND	32.6	ND
8.8	ND	ND	ND	33.8	ND
9	4.0	12.1	9.0	32.0	40.0
9.5	ND	15.1	10.5	34.0	38.7
9.8	5.0	ND	21.2	ND	ND
9.9	ND	ND	ND	34.6	32.0
10	ND	28.7	ND	ND	ND

Abbreviation: GHM, Grosses Heiliges Meer.

The copy number of the 16S rRNA gene was retrieved from all available cyanobacterial genomes in NCBI and is summarized by order in Table A3.

**Table A3:** Copy number of 16S rRNA gene.

	Order				
	Chroococcales	Gloeobacterales	Nostocales	Oscillatoriales	Synechococcales
Mean	2.3	1.0	4.0	3.0	2.0
Std. dev.	0.5	0.0	0.9	1.0	0.7

Abbreviation: rRNA, ribosomal RNA.

Lake water for fluorescent cell counts was preserved with 2.5% paraformaldehyde and stored at 4°C. Samples were washed, sonicated, iron minerals were digested if necessary, and stained with Sytox according to procedures described in Wu et al. (2014). Imaging was performed on a Leica DM5500B fluorescent microscope. Counts were made on a single filter per depth. At least 400 cells were counted per sample, averaging about 12 fields of view per sample. Counts were not performed in September 2018. Counts did not distinguish between bacteria, phytoplankton, or zooplankton and thus cannot be quantitatively related to biomass.
